# Bisphenol-A reduces DNA methylation after metabolic activation

**DOI:** 10.1186/s41021-022-00249-y

**Published:** 2022-07-25

**Authors:** Kei-ichi Sugiyama, Mawo Kinoshita, Petr Grúz, Toshio Kasamatsu, Masamitsu Honma

**Affiliations:** 1 Division of Genetics and Mutagenesis, National Institute of Health Sciences, 3-25-26 Tonomachi, Kawasaki-ku, Kawasaki-shi, Kanagawa 210-9501 Japan; 2 Division of General Affairs, National Institute of Health Sciences, 3-25-26 Tonomachi, Kawasaki-ku, Kawasaki-shi, Kanagawa 210-9501 Japan

**Keywords:** Bisphenol-A, DNA methylation, Metabolic activation, Yeast, *FLO1*

## Abstract

Bisphenol-A (BPA) is an important environmental contaminant with adverse health effects suspected to be mediated through epigenetic mechanisms. We had reported that the FLO1-dependent flocculation of transgenic yeast expressing human DNA methyltransferase (*DNMT* yeast) is a useful tool in epigenotoxicology studies. In this report, we have investigated the effects of BPA in the presence of metabolic activation (S-9 mix) on the transcription level of the *FLO1* gene in the *DNMT* yeast. In the presence of metabolic activation, BPA inhibited the intensity of green fluorescence reporter protein (GFP) driven by the *FLO1* promoter. A metabolite of BPA, 4-methyl-2,4-bis(p-hydroxyphenyl) pent-1-ene (MBP), also exhibited similar inhibitory effect. Furthermore, BPA in the presence of S-9 mix had only a weak while MBP had no inhibitory effects on the expression of modified GFP reporter gene under the control of *FLO1* promoter with reduced CpG motifs. Aforementioned behavior was confirmed by the inhibition of flocculation as well as *FLO1* gene mRNA expression. In addition, the global DNA methylation level in the human HEK293 cells was also reduced by MBP. These results indicate that BPA metabolites have inhibitory effect on DNA methylation. Our approach offers a novel *in vitro* method for screening for chemicals that can alter the epigenome by a mechanism dependent on their metabolic activation.

**Supplementary Information:**

The online version contains supplementary material available at 10.1186/s41021-022-00249-y.

## Introduction

Bisphenol-A (BPA) is commonly used in the manufacturing of polycarbonate plastics and epoxy resins and is known to have weak estrogenic activity [1]. Since human exposure to BPA is wide-spread, special attention has been paid to its detrimental effects. Exposure to BPA has been shown to induce several epigenetic modifications in both animal and human cells [2, 3]. However, most of the evidence on these epigenetic modifications were obtained by investigating only BPA itself.

Yoshihara et al. have reported that *in vitro* estrogenic activity of BPA was elevated after incubation with rat liver S9 fraction in the presence of an NADPH-generating system (S-9 mix), and then, identified the active metabolite as 4-methyl-2,4-bis(4-hydroxyphenyl) pent-1-ene (MBP) [4, 5]. Further it has been shown that MBP has more potent estrogenic activity than BPA both *in vitro* and *in vivo* [4–6]. These reports suggest that metabolites need to be taken into account for the BPA detrimental effect assessment. Nevertheless, little is known about a role of metabolites in the epigenetic modifications induced by BPA.

We previously reported that a yeast strain transformed with a plasmid encoding human DNA methyltransferase *DNMT1* and *DNMT3B* cDNAs gained a flocculation phenotype, which was suppressed with DNMT inhibitors [7]. We also determined that the flocculation level of the *DNMT* yeast which was transformed with the *DNMT* genes as well as control strain was influenced by histone modification or the chromatin structure state [8]. In addition, the flocculation of the yeast correlated with the expression of *FLO1*, which encodes a cell wall surface protein that promotes cell–cell adhesion [7–9]. Thus, the levels of flocculation and *FLO1* expression are potentially useful biomarkers for studying epigenetic effects [10–12].

S-9 mix utilized in the investigations by Yoshihara et al. has been widely used for *in vitro* genotoxicity tests, such as the Ames test or *in vitro* chromosomal aberration test, to detect metabolically activated mutagens [13, 14]. In the present study, we examined the effect of BPA, suspected epigenetic modifier, on the transcription level of *FLO1* in the presence or in the absence of metabolic activation (± S-9 mix). Further we have also assessed the global DNA methylation level in human cell line HEK293.

## Materials and methods

### Strains, culture conditions and chemicals

The *Saccharomyces cerevisiae* strain YPH250 used in this study and reffered to as the *DNMT* yeast optionally carrying the plasmids pF1GS or pF1GSTpA has been described in details previously [11]. All the plasmids and strains used in this strain are summarized in Tables 1 and 2. The yeast cell cultivation conditions were essentially the same as described previously with the exception that we used non-shaking condition for cultures with the added S-9 activation mix [11].

**Table 1:** Plasmids used in the study

Plasmid	Description	Reference
pY2CThD1	pYES2/CT harboring-human *DNMT1* cDNA	[7]
pY3CThD3B	pYES3/CT harboring-human *DNMT3B* cDNA	[7]
pF1GS	*FLO1-GFP*. Parent: p313eGFP	[10]
pF1GSTpA	CpG reduced *FLO1-GFP*. Parent: p313eGFP	[10]
p313eGFP	pRS313 (*CEN*-type, *HIS3* marker) carrying a GFP variant	[9]

**Table 2:** Yeast strains used in the study

Strain	Genotype	Plasmid	Name
YPH250	*MATα trp1-∆1 his3-∆200 leu2-∆1 lys2-801 ade2-101 ura3-52*	pY2CThD1, pY3CThD3B	*DNMT* yeast
YPH250	*DNMT* yeast	pF1GS	
YPH250	*DNMT* yeast	pF1GSTpA	
YPH250	*DNMT* yeast	p313eGFP	

BPA, bisphenol-A (CAS. No. 80–05-7) was purchased from Wako Pure Chemical Industries (Osaka, Japan) and MBP, 4-methyl-2,4-bis(p-hydroxyphenyl) pent-1-ene (CAS. No. 13464–24-9), was purchased from Organochem Ltd. (Budapest, Hungary). Rat liver S9 fraction mixed with co-factor mixture (S-9 mix, product name: “Frozen S9MIX for Ames test”) was purchased from IEDA TRADING CORPORATION (Tokyo, Japan).

### *FLO1* promoter-based green fluorescent protein (GFP) reporter gene assay

The reporter gene assay has been performed according to our previously established protocol [9]. Briefly, the *DNMT* yeast cells bearing a reporter plasmid (Table 1) were cultured for 24–25 h after adding 200 μl of solution containing each chemical plus S-9 mix or 0.2 mM sodium phosphate (NaP) buffer (pH7.4) for the control in total 1.5 ml of SD minimal medium to early stationary phase. Each chemical was preincubated with the S-9 reagent at 37 °C for 20 min before adding it to the cells. The maximum test concentration was set around the growth-arrest dose. 0.6 ml aliquotes of the treated cell cultures were collected by centrifugation and further processed essentially the same as described previously [11] to obtain the fluorescence values.

### Measurement of flocculation

The *DNMT* yeast cells grown in the presence of varying concentrations of BPA with the S-9 mix (see above) to an early stationary phase were lightly vortexed in test tubes, allowed to settle for 30 min and then photographed in the horizontal aspect and from beneath. The diameters of both the floc (F) and the tube (T) were measured and the relative flocculation activity was calculated using the equation as before [11]:

$$\mathrm{Relative}\;\mathrm{flocculation}\;\mathrm{activity}\:=\:100\:\times\:(\mathrm F/\mathrm T)$$

### Semi-quantitative reverse transcription-polymerase chain reaction

The *DNMT* yeast cells grown with or without S-9 mix with varying concentrations of test chemicals (see above) for 48 h were harvested by centrifugation. Then the total RNA was extracted by a glass bead approach using the RNeasy kit (Qiagen N.V., Venlo, Limburg, The Netherlands) followed by RNase-free DNase treatment, according to the manufacturer’s instructions. The total isolated RNA (0.05 μg) was subjected to reverse transcription-polymerase chain reaction (RT-PCR) with the SuperScript® One-Step RT-PCR System with Platinum® Taq DNA Polymerase (Invitrogen). The primers used for the reaction were the same as described previously [11]: *FLO1*, 5′-CTCATCGCTATATGTTTTTGG-3′(forward) and 5′-CGAGTAAACAACCTTCATTGG-3′ (reverse); *ACT1*, 5′-ATTCTGAGGTTGCTGCTTTGG-3′ (forward) and 5′-GAAGATTGAGCAGCGGTTTGC-3′ (reverse).

### Tetrazolium (MTT) assays of cell viability

A total of 1 × 10^4^ HEK293 cells in 100 μL of Dulbecco’s modified Eagle’s medium (DMEM) was plated onto 96-well plate and was cultivated at 37 °C in a humidified atmosphere with 5% CO_2_. After cultivation for 24 h, culture medium was replaced with DMEM containing MBP at 5–80 µM or 0.1% ethanol (control) and cells were grown for additional 48 h. Cell viability was then assessed using MTT assays (Roche Diagnostics, Mannheim, Germany) according to the manufacturer’s instructions.

### Detection of 5-methylcytosine (5mC) using enzyme linked immunosorbent assay (ELISA)

The contents of 5-methylcytosine (5mC) in genomic DNA of HEK293 cells was measured using MethylFlash Global DNA Methylation (5-mC) ELISA Easy Kit (Colorimetric; Epigentek Group Inc., NY, USA) according to the manufacturer’s instructions. The HEK293 cells were seeded into 6-well plates at a density of 2.0 × 10^5^ cells/well in 2 mL of culture and were cultured at 37 °C. After 24 h, the medium was replaced with DMEM containing 20, 40 µM MBP or 0.1% ethanol (control) and the cells were further cultivated for 48 h before proceeding with preparation of genomic DNA. 100-ng genomic DNA isolated using QIAamp DNA Mini Kits (Qiagen) was used to quantify 5mC by measuring absorbance of capture and detection antibodies at 450 nm with a Multiskan GO plate reader (Thermo). Data are presented as percentage of methylated DNA (5mC%).

### Statistical analysis

All the statistical tests comparing multiple groups were performed as described previously using the ANOVA and Dunnett’s post hoc tests [11] and our data are presented as the means ± standard errors (SEM).

## Results

### Effect on FLO1 reporter activities

**Fig. 1:**
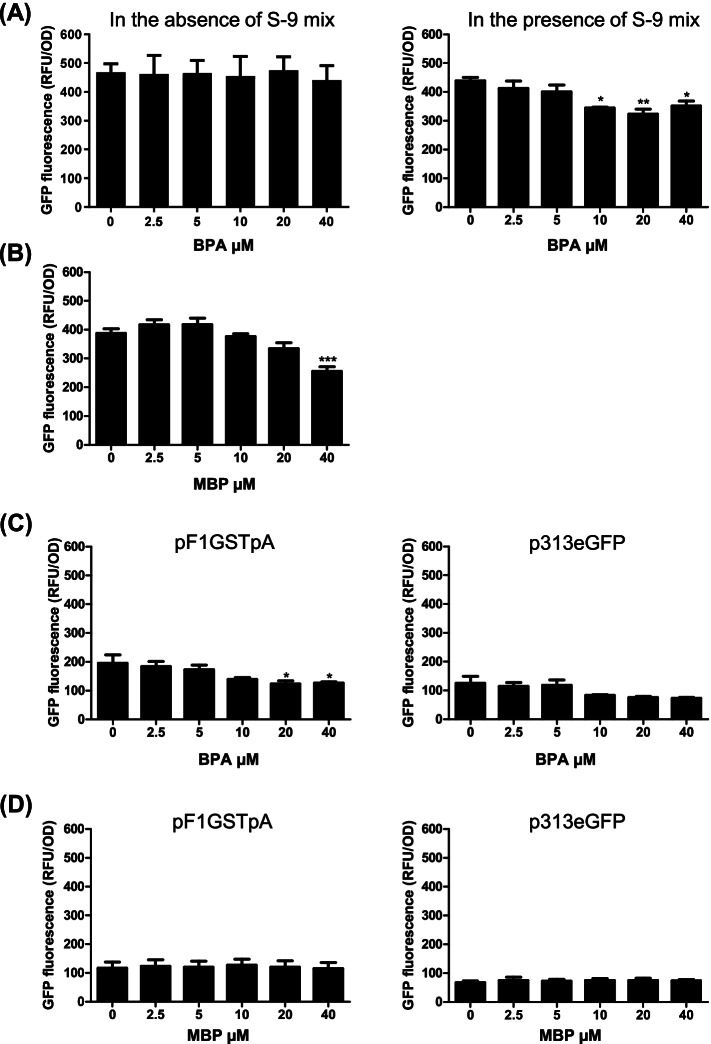
Effect of BPA and MBP on the GFP fluorescence levels driven by the *FLO1* promoter. **A** *DNMT* yeast transformed with pF1GS was grown at the indicated concentrations of BPA in the absence of S-9 mix to OD600 of 1.6–2.7 or in the presence of S-9 mix to OD600 of 2.6–3.2. Then, the intensity of GFP was measured. **B** *DNMT* yeast transformed with pF1GS was grown at the indicated concentrations of MBP to OD600 of 1.0–2.6 in the absence of S-9 mix. Then, the intensity of GFP was measured. **C** *DNMT* yeast transformed with pF1GSTpA or p313eGFP was grown at the indicated concentrations of BPA in the presence of S-9 mix to OD600 of 2.0–3.0 (*DNMT* yeast harboring pF1GSTpA) or to OD600 of 2.0–3.1 (*DNMT* yeast harboring p313eGFP). Then, the intensity of GFP was measured. **D** *DNMT* yeast transformed with pF1GSTpA or p313eGFP was grown at the indicated concentrations of MBP in the absence of S-9 mix to OD600 of 1.1–2.7 (*DNMT* yeast harboring pF1GSTpA) or to OD600 of 0.9–1.9 (*DNMT* yeast harboring p313eGFP). Then, the intensity of GFP was measured. The data are presented as the means ± SEM from more than three independent experiments. Statistical analysis was performed using ANOVA, followed by Dunnett’s post hoc test. (*P < 0.05, **P < 0.01, ***P < 0.001 versus control)

During the first analysis, we examined effects of BPA on the GFP reporter gene activity under control of the *FLO1* promoter in the presence or in the absence of metabolic activation (± S-9 mix). Further, the effect of MBP, a metabolite of BPA, was also examined in the absence of metabolic activation. As shown in Fig. 1A, BPA (10–40 µM) significantly decreased the GFP fluorescence driven by the *FLO1* promoter in *DNMT* yeast in the presence of metabolic activation (+ S-9 mix), but not in the absence of metabolic activation (-S-9 mix). This effect depended on the S-9 mix enzymatic activity and was not caused by mere rat liver S9 fraction since the inactive S-9 mix lacking the NAD(P)H co-factors was ineffective (Fig. s1). Furthermore, as shown in Fig. 1B, 40 µM MBP significantly decreased the GFP fluorescence in the absence of metabolic activation (-S-9 mix). These results suggest that BPA was metabolized to exhibit inhibitory effect on the *FLO1* promoter activity. The mRNA levels of *FLO1* are upregulated in *DNMT* yeast with higher DNA methylation level of *FLO1* promoter [10].

Then, we examined effect of BPA with S-9 mix and MBP on the reporter activity controlled by some CpG sites replaced with TpA in *FLO1* promoter in *DNMT* yeast transformed with pF1GSTpA as well as by *DNMT* yeast carrying empty vector (p313eGFP). As shown in Fig. 1C and D, the basal fluorescence intensities in *DNMT* yeast with pF1GSTpA or p313eGFP were lower than that of the native *FLO1* promoter in *DNMT* yeast. BPA with S-9 mix showed weak but MBP had no inhibitory effect on GFP intensity driven by the CpG-reduced *FLO1* promoter in *DNMT* yeast. Neither BPA with S-9 mix nor MBP decreased the GFP fluorescence in *DNMT* yeast carrying p313eGFP. Since MBP only decreased intensities of GFP expression driven by native or CpG-reduced *FLO1* promoter, implying that MBP interferes with *FLO1* promoter-driven reporter activities through the CpG motif on the promoter.

### Effect on flocculation

**Fig. 2:**
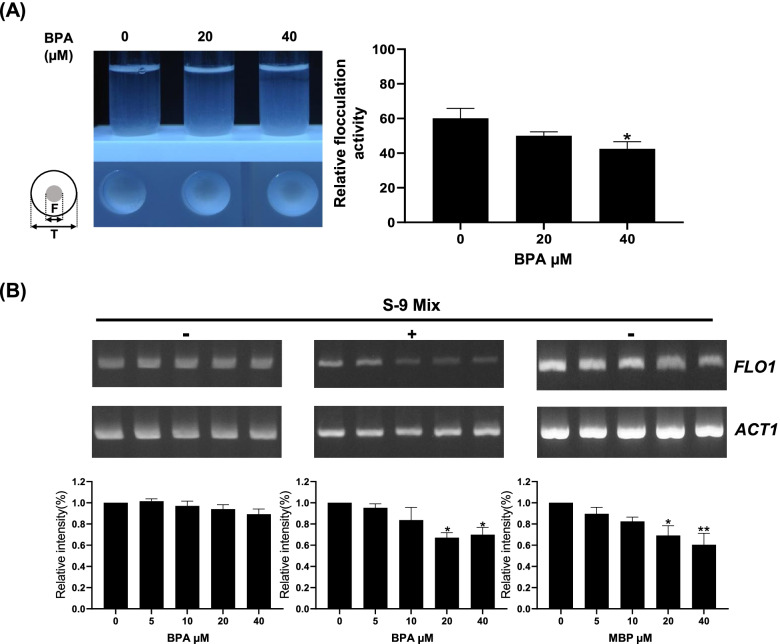
Effect of BPA in the presence of S-9 mix on the flocculation of *DNMT* yeast. **A** *DNMT* yeast was grown at the indicated concentrations of BPA to OD600 of 3.3-3.7 in the presence of S-9 mix. The pictures were taken after allowing the agitated culture to settle at room temperature for 30 min. This experiment for measuring relative flocculation activity was repeated at least three times with similar results and the data from one representative experiment is shown. The histogram shows the relative flocculation activity. The data are presented as the means ± SEM from more than three independent experiments. Statistical analysis was performed using ANOVA, followed by Dunnett’s post hoc test. (*P < 0.05 versus control). **B** *DNMT* yeast was grown at the indicated concentrations of BPA (± S-9 mix) or MBP. Total RNA was extracted from the yeast cells, and the mRNA levels of *FLO1* and *ACT1* were determined by semi-quantitative RT-PCR. The results are representative of at least three independent experiments. The histograms show the relative intensity of *FLO1* mRNA levels normalized to the levels of *ACT1* mRNA and the control was set as 1.0. The data are presented as the means ± SEM from more than three independent experiments. Statistical analysis was performed using ANOVA, followed by Dunnett’s post hoc test. (*P < 0.05, **P < 0.01 versus control).

It was found that the activity of the *FLO1*-GFP reporter was reduced by BPA (+ S-9 mix) and MBP (Fig. 1A and B). To confirm this suppressive effect, we next examined the effect of BPA (+ S-9 mix) on the yeast flocculation. It has been previously reported that *DNMT* yeast displays inducible flocculation phenotype [7]. As shown in Fig. 2A, under the control condition, *DNMT* yeast which enhances the basal level expression of *FLO1* gene showed a high relative flocculation activity of over 70%. On the other hand, at 20–40 µM, BPA (+ S-9 mix) caused a dose-dependent reduction in the flocculation of *DNMT* yeast. These results suggest that BPA metabolites can repressed flocculation in *DNMT* yeast via reduction of *FLO1* transcription.

### Effect on FLO1 mRNA levels

To examine whether BPA (± S-9 mix) or MBP suppresses *FLO1* expression at the mRNA level in *DNMT* yeast, we performed RT-PCR analysis for *FLO1*. In the 5–40 µM concentration range, *FLO1* mRNA levels in *DNMT* yeast treated with BPA (+ S-9 mix) and MBP, but not BPA (-S-9 mix), decreased in a dose-dependent manner compared with the untreated control (Fig. 2B). These results are consistent with the effect of BPA (± S-9 mix) and MBP on *FLO1* promoter-driven GFP reporter activity of *DNMT* yeast (Fig. 1A and B) and indicate that transcription of the endogenous *FLO1* gene is downregulated by BPA treated with S-9 mix and MBP.

### Effect of MBP on the methylation levels of genomic DNA from HEK293 cells

**Fig. 3:**
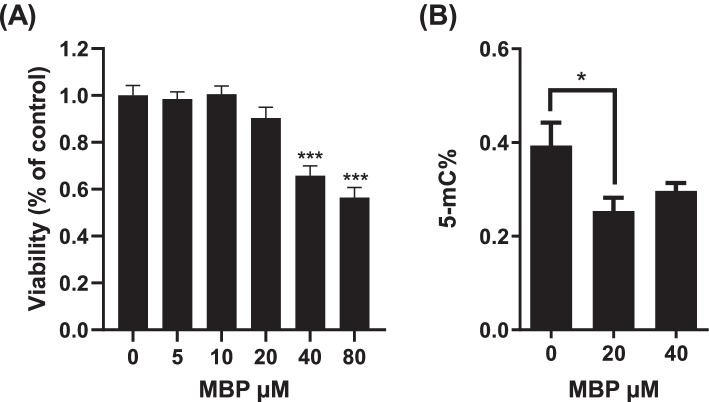
Effects of MBP on cell viability and DNA methylation levels in HEK293 cells. **A** Cell viability of HEK293 cells was determined after cultivating them in DMEM containing the indicated concentrations of MBP for 48 h. Data are expressed as mean % viability values ± SEM of four independent experiments. **B** DNA methylation levels in HEK293 cells were quantified using ELISA after 48-h treatments with 20 and 40 µM MBP. Data are presented as means ± SEM from three independent experiments. Statistical analysis was performed using ANOVA, followed by Dunnett’s post hoc test. (*P < 0.05, ***P < 0.001 versus control).

To evaluate the effect of MBP on DNA methylation in mammalian cells, we analyzed 5mC levels in genomic DNA extracted from HEK293 cells treated with MBP using ELISA. As shown in Fig. 3A, MBP at concentrations of 40 and 80 µM significantly reduced cell viability. Next, we selected high dose of MBP at 40 µM in the ELISA and investigated the global 5mC levels in HEK293 treated with 20 and 40 µM MBP. 5mC% contents of the cells in the presence of 20-µM MBP was significantly decreased as compared with control cells (Fig. 3B), suggesting that MBP exposure has the potential to reduce the level of 5mC in genomic DNA of HEK293 cells.

## Discussion

In the present study, we examined the effect of BPA in the presence or in the absence of metabolic activation (± S-9 mix) on the transcription level of *FLO1*. As a result, BPA (+ S-9 mix) and MBP, a metabolite of BPA, showed inhibitory effect on GFP fluorescence driven by the *FLO1* promoter suggesting that BPA was metabolized to MBP to exert inhibitory activity on DNA methylation. On the other hand, BPA (+ S-9 mix) had only a weak inhibitory effect while MBP had no effect on GFP fluorescence driven by the modified *FLO1* promoter with reduced CpG motifs (Fig. 1A and B). This suggests that both BPA (+ S-9 mix) and MBP interfere with DNA methylation mechanisms through the CpG motifs in the *FLO1* promoter and the slight difference observed between them can be attributed to other metabolites of BPA. The BPA inhibitory effect on DNA methylation in the presence of metabolic activation was also confirmed by the inhibition of the flocculation as well as by its suppressive effect on the *FLO1* mRNA expression in *DNMT* yeast (Fig. 2).

Many studies have reported that change of DNA methylation level is involved in the detrimental effects of BPA [2, 3]. For example, Dolinoy et al. reported that BPA affected DNMTs in viable yellow agouti mice, which carry a mutant allele with a retrotransposon (intracisternal A particle; IAP) inserted upstream of the Agouti gene that defines hair color. They examined the hair color of the litters of mice treated with BPA and reported that BPA promoted DNA demethylation of IAP, which induced the homeostatic expression of Agouti and shifted the hair color toward yellow [15]. On the other hand, another group conducted a large-scale validation experiment and reported that they could not reproduce the results [16]. It has been also reported that BPA exposure during fetal mouse growth promoted neuronal differentiation and migration, suggesting that it is accompanied by changes in DNA methylation [17]. It seems that there is no consensus in the effect of BPA on DNA methylation yet. Our results suggest that metabolic activation plays a key role in the effect of BPA on DNA methylation and provide some evidence for understanding the biological effects of BPA. Furthermore, we were able to assess the transcription level of *FLO1* under the metabolic activation condition. We also observed a decreasing trend in 5mC level in HEK293 cells treated with MBP, which was not associated with cell death (Fig. 3), implying that BPA metabolites as well as MBP may influence global DNA methylation level in mammalian cell.

It has been difficult to evaluate the effect of metabolites on epigenetic modifications. To our knowledge, there is currently no report on metabolically activated epi-mutagens. Therefore, our present study expands the applicability of an *in vitro* experimental system for studying the epigenetic effects of various substances under the metabolic activation condition.

## Conclusions

The epigenetic effects of BPA and related substances in the presence or absence of metabolic activation (± S-9 mix) were investigated using the flocculation levels and expression of flocculation**-**related gene *FLO1* in *DNMT* yeast as an indicator. BPA (+ S-9 mix) as well as MBP, a metabolite of BPA, inhibited the intensity of GFP fluorescence driven by the *FLO1* promoter. This phenomenon has been confirmed by the inhibition of flocculation and by the suppressive effect on the *FLO1* mRNA expression in *DNMT* yeast. Our results thus indicate that BPA can be metabolized to exert inhibitory effects on DNA methylation. This innovative approach offers useful *in vitro* method for screening similar chemicals that exhibit epigenetic effect via metabolic activation.

## Supplementary Information

**Additional file 1: Fig. S1.** Effect of BPA using the enzymatically active and inactive S-9 mixes on the fluorescence levels of GFP driven by the *FLO1* promoter in the *DNMT* yeast transformed with pF1GS.

**Additional file 2.**

## Data Availability

Not applicable.

## Abbreviations

BPA: Bisphenol-A
MBP: 4-Methyl-2,4-bis(p-hydroxyphenyl) pent-1-ene
DNMT: DNA methyltransferase
GFP: Green fluorescence reporter protein
RT-PCR: Reverse transcription-polymerase chain reaction
DMEM: Dulbecco’s modified Eagle’s medium
5mC: 5-Methylcytosine
ELISA: Enzyme linked immunosorbent assay
