# Supplementary figures and images for: Bisphenol-A reduces DNA methylation after metabolic activation
Source: Genes Environ. 2022 Jul 25;44:20. doi: 10.1186/s41021-022-00249-y (PMC9316663; doi:10.1186/s41021-022-00249-y)

## Slide 1
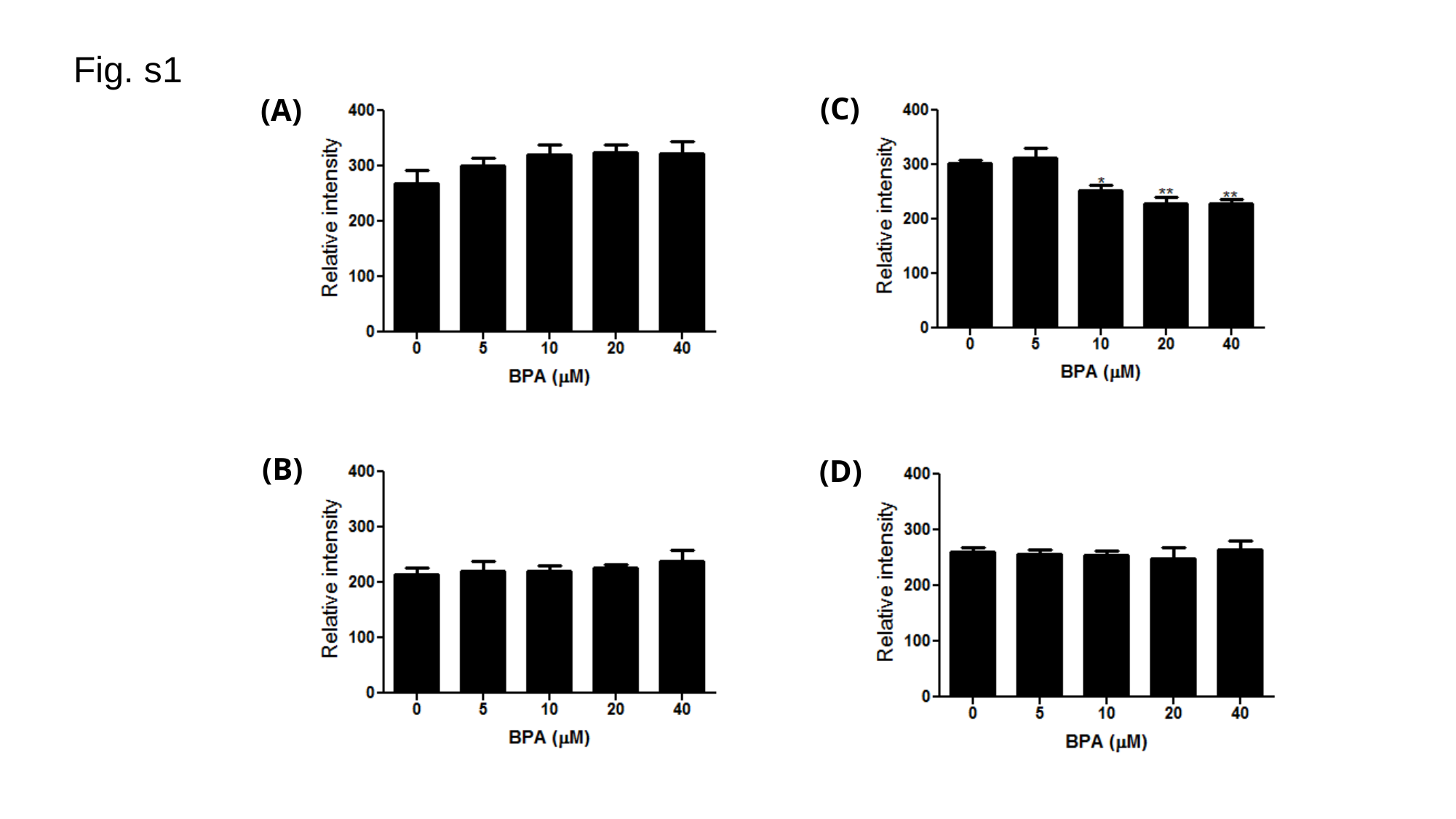

Fig. s1
(C)
(A)
(B)
(D)

Supplement: Supplementary file 1 — Additional file 1: Fig. S1. Effect of BPA using the enzymatically active and inactive S-9 mixes on the fluorescence levels of GFP driven by the FLO1 promoter in the DNMT yeast transformed with pF1GS. [file 41021_2022_249_MOESM1_ESM.pptx]
