# Supplementary material for: Bisphenol-A reduces DNA methylation after metabolic activation
Source: Genes Environ. 2022 Jul 25;44:20. doi: 10.1186/s41021-022-00249-y (PMC9316663; doi:10.1186/s41021-022-00249-y)
Supplement: Supplementary file 2 — Additional file 2. [file 41021_2022_249_MOESM2_ESM.doc]

1. Supplementary materials & methods
   1. Strains, culture conditions and chemicals

The *DNMT* yeast transformed with the plasmid pF1GS were cultivated under non-shaking condition at 30 °C in a synthetic dextrose (SD) minimal medium containing 2% of glucose, 0.67% of yeast nitrogen base and appropriate amino acids. Rat liver S9 microsomal fraction was the product of Oriental Yeast Co., LTD (Shiga, Japan) and has been optionally mixed with the co-factor solution containing glucose, NADPH and NADH prior to use as per the manufacturer’s instructions.

- 1. *FLO1* promoter-based green fluorescent protein (GFP) reporter gene assay

The reporter gene assay was performed as described previously [9]. Briefly, the *DNMT* yeast cells bearing pF1GS were cultured for 24–25 h after adding 200 μl of solution containing BPA plus full S-9 mix (Rat liver S9 microsomal fraction and the co-factor solution), Rat liver S9 microsomal fraction only, the co-factor solution only or 0.2 mM Nap buffer (pH7.4) in total 1.5 ml of SD minimal medium to early stationary phase. BPA was preincubated with each reagent at 37 °C for 20 min before mixing it with cells. Cells from 0.6 ml cultures were collected by centrifugation, the cell pellet was washed, and resuspended in 0.6 ml of 0.9% NaCl. Fluorescence measurements were performed using 100 μl of sample in black flatbottomed plate (Sterilin Sero-Well, UK) with a microplate fluorometer (TriStar2 LB 942; Berthold Technologies GmbH & Co. KG, Bad Wildbad, Germany). Fluorescence intensity (excitation wavelength at 485 nm and emission wavelength at 535 nm) was measured to estimate the relative levels of GFP expression. Background fluorescence, as calculated from a well containing 100 μl of 0.9% NaCl, was subtracted from the measurements, and the fluorescence intensity was normalized to OD600 = 1.0.

Supplementary figure legend

Fig. s1

Effect of BPA using the enzymatically active and inactive S-9 mixes on the fluorescence levels of GFP driven by the *FLO1* promoter in the *DNMT* yeast transformed with pF1GS.

(A) *DNMT* yeast was grown in the presence of the indicated concentrations of BPA to OD600 of 1.7-4.5 (in the absence of Rat liver S9 microsomal fraction and co-factor solution) and then the intensity of GFP was measured.

(B) *DNMT* yeast was grown in the presence of the indicated concentrations of BPA to OD600 of 2.7-4.6 (in the presence of co-factor solution only) and then the intensity of GFP was measured.

(C) *DNMT* yeast was grown in the presence of the indicated concentrations of BPA to OD600 of 2.7-4.6 (in the presence of S-9 mix which contains Rat liver S9 microsomal fraction and the co-factor solution) and then the intensity of GFP was measured.

(D) *DNMT* yeast was grown in the presence of the indicated concentrations of BPA to OD600 of 2.6-4.4 (in the presence of Rat liver S9 microsomal fraction only) and then the intensity of GFP was measured.

The data are presented as the means ± SEM from more than three independent experiments. Statistical analysis was performed using ANOVA, followed by Dunnett’s *post hoc* test. (**P*<0.05, ***P*<0.01 versus control).
